# Homing of radiolabelled xenogeneic equine peripheral blood-derived MSCs towards a joint lesion in a dog

**DOI:** 10.3389/fvets.2022.1035175

**Published:** 2022-11-24

**Authors:** Charlotte Beerts, Glenn Pauwelyn, Eva Depuydt, Yangfeng Xu, Jimmy H. Saunders, Kathelijne Peremans, Jan H. Spaas

**Affiliations:** ^1^Boehringer Ingelheim Veterinary Medicine Belgium, Evergem, Belgium; ^2^Department of Morphology, Imaging, Orthopedics, Rehabilitation and Nutrition of Domestic Animals, Faculty of Veterinary Medicine, Ghent University, Merelbeke, Belgium; ^3^Department of Surgery and Anaesthesiology of Domestic Animals, Faculty of Veterinary Medicine, Ghent University, Merelbeke, Belgium; ^4^Ghent Experimental Psychiatry (GHEP) Laboratory, Department of Head and Skin, Faculty of Medicine and Health Sciences, Ghent University, Ghent, Belgium; ^5^Boehringer Ingelheim Animal Health, Athens, GA, United States

**Keywords:** mesenchymal stem cell, canine, cranial cruciate ligament tear, homing, radiolabelling

## Abstract

Osteoarthritis (OA) is a highly prevalent condition in dogs, causing a substantial reduction in quality of life and welfare of the animals. Current disease management focusses on pain relief but does not stop the progression of the disease. Therefore, mesenchymal stem cells (MSCs) could offer a promising disease modifying alternative. However, little is known about the behavior and the mode of action of MSCs following their administration. In the current case report, ^99m^Technetium labelled xenogeneic equine peripheral blood-derived MSCs were intravenously injected in a 9 year old dog suffering from a natural occurring cranial cruciate ligament rupture. The biodistribution of the MSCs was evaluated during a 6-h follow-up period, using a full body scintigraphy imaging technique. No clinical abnormalities or ectopic tissue formations were detected throughout the study. A radiopharmaceutical uptake was present in the liver, heart, lung, spleen, kidneys and bladder of the dog. Furthermore, homing of the radiolabelled MSCs to the injured joint was observed, with 40.61 % higher uptake in the affected joint in comparison with the healthy contralateral joint. Finally, a local radioactive hotspot was seen at a part of the tail of the dog that had been injured recently. The current study is the first to confirm the homing of xenogeneic MSCs to a naturally occurring joint lesion after IV administration.

## Introduction

Osteoarthritis (OA) is a highly prevalent condition in dogs, causing a substantial reduction in quality of life and welfare of the animals. In dogs, secondary osteoarthritis, caused by an underlying disease process or an injury (i.e., cranial cruciate ligament disease, hip or elbow dysplasia), is more common than primary osteoarthritis (1). Current disease management involves conservative treatments [i.e. (non-) steroidal inflammatory drugs and opioids] focusing on providing relief of the disease symptoms, but they do not delay or stop the disease progression, let alone reverse it. Surgical management comprises total joint replacement and arthrodesis which are highly invasive procedures (2).

Mesenchymal stem cell (MSC) therapies have been studied for the past decade and could offer a promising alternative to the currently proposed treatments. The beneficial effects of MSCs such as differentiation in chondrocytes and immunomodulation, could provide a more durable solution for the treatment of OA, with a potential impedance or stop of the disease progression, and might even revert the sustained damage or prevent the development of OA (3, 4).

Different reviews have described the safety of autologous MSCs harvested from bone marrow or fat for local intra-articular application (5, 6). However, autologous MSCs must be harvested from the patients itself, cultured for several weeks and administered back to the patient. This often involves an invasive procedure for the already compromised patient and does not allow immediate treatment of an acute flare. Moreover, the quality of MSCs declines with increasing age (7, 8), which constitutes a problem since OA becomes more prevalent with age. Therefore, allogeneic and xenogeneic MSC therapy can be an interesting alternative. The absence of major histocompatibility complex class II expression on the cell surface markers of MSCs allows the allogeneic and xenogeneic transplantation without the induction of immunological rejection (9). Furthermore, xenogeneic stem cell therapy using equine MSCs can be of a great interest thanks to the absence of transmission of species-specific diseases and because of the relatively lower culture capacity of canine MSCs (6). The latter implicates that limited passaging of the canine MSCs is possible. In contrast, considerably more passaging steps are possible with equine MSCs resulting in less blood collections and increased production volumes (10). Additionally, blood is minimally invasive, easier to harvest, and larger amounts of blood can be sourced with from horses than from dogs no or little donor site morbidity. The safe administration of porcine MSCs in dogs was reported by Tsai et al. reported (11) and our group recently described the safe use of an intra-articular injection of equine peripheral blood-derived mesenchymal stem cells (ePB-MSCs) in dogs suffering from naturally occurring OA (12). However, this was a feasibility study and more research is needed to investigate the safety and efficacy of ePB-MSCs for the treatment of OA in dogs. The systemic injection of MSCs would ease their use in practice and could create a more suitable option for treating patients suffering from OA in multiple joints. Additionally, the systemic administration of MSCs could induce a stronger interaction with the immune system resulting in systemic anti-inflammatory and immunomodulatory effects (13, 14).

The evaluation of the biodistribution of MSCs following their intravenous administration in dogs suffering from non-infectious joint lesions would help to better understand the behavior and mode of action of the MSCs. Recently, Barthélémy et al. (15) reported the scintigraphic tracking of myogenic stem cells in the Golden Retriever Muscular Dystrophy model using a ^111^In-oxine labelling technique. In this study, following their injection in the femoral artery, the myogenic stem cells were mainly trapped in the injected limb and the lung. Our group recently reported the tracking of radiolabelled ePB-MSCs following intravenous, intramuscular and subcutaneous administration in healthy dogs. In that study, radiopharmaceutical uptake was predominantly seen in the heart, lung, liver and bladder (16). To the author's knowledge, no study has reported the tracking of MSCs in dogs suffering from orthopedic lesions. Therefore, the goal of this study was to evaluate the biodistribution of radiolabelled ePB-MSCs following intravenous injection in a dog with a naturally occurring cranial cruciate ligament rupture during a 6 h follow-up period, using a full body scintigraphy imaging technique.

## Materials and methods

### Experiment

In this experiment, the biodistribution of intravenously administrated ePB-MSCs was evaluated in a dog with a naturally occurring cranial cruciate ligament rupture.

### Animals

The blood collection of the donor horse and treatment administration (approval number: EC_2016_003, EC_2021_002) was approved by an ethics committee with independent members evaluating the application as approved by the Flemish government (permit number: LA1700607). Additionally, a deontological approval was obtained for the treatment administration in the dog (DWZ-KF-21-1.15-54). All animal handlings were conducted according to European, national and regional animal welfare regulations (Directive 2001/82/EC as amended, Belgian animal welfare legislation (KB 29/05/2013), Directive 2010/63/EU and EMEA/CVMP/816/00-Final). A written owner informed consent was obtained prior to study start.

**Figure 1:**
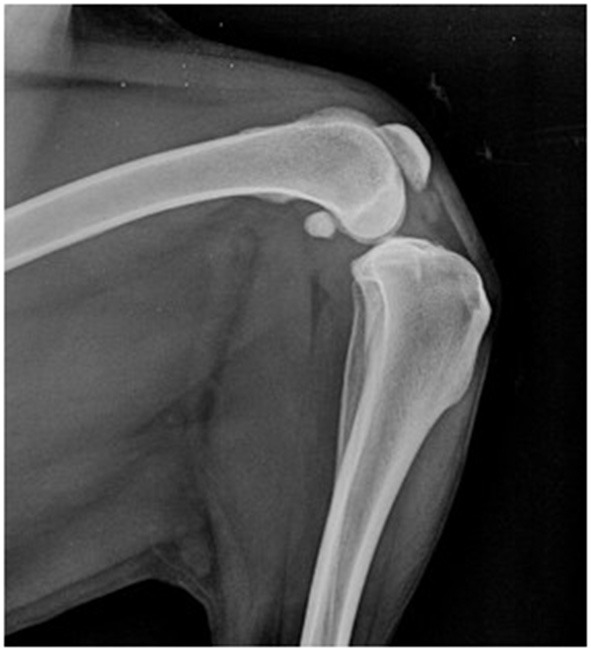
Lateral view of the left stifle joint under tibial compression test. A compression of the infra-patellar fat pad, indicating synovitis, is observed and a cranial displacement of the tibia relative to the femur is seen.

The patient included in the experiment was a 9-year-old neutered female of mixed breed (Border collie x Labrador) with a complete rupture of the cranial cruciate ligament that was diagnosed and confirmed by radiographic assessment (Figure 1) within 3 weeks before the start of the experiment.

A general physical examination was performed on the study day and on the next day to assess the following parameters: rectal temperature, respiratory rate, heart rate, mucosal membranes, capillary refill time, body conditions score, mentation and hydration.

### Collection and cultivation of ePB-MSCs

The ePB-MSCs were GMP-manufactured in a GMP-certified site (number: BE/GMP/2018/123) as earlier described by our group (17). Briefly, the MSCs were isolated after blood was taken from the jugular vein of a donor horse (approval number EC: EC_2012_001 and 2016_003). An analysis of the serum for a variety of infectious diseases was performed by Böse laboratory (Harsum, Germany). As previously described by our group (10), the blood was centrifuged and the buffy coat was collected. The ePB-MSCs were washed, cultivated until passage five and characterized for viability, morphology, presence of cell surface markers and population doubling time. The ePB-MSCs were then frozen as an intermediate cell stock. Once characterisation was accomplished, the intermediate cell stock was thawed and cultivated until passage 10 before being trypsinized, resuspended in DMEM, filtered twice through a 40 μm filter and vialed at 0.3 x 10^6^ cells/mL in a mixture of DMEM supplemented with 10% dimethylsulfoxide (DMSO). The vials were stored at−80°C until further use.

### ^99m^Tc-labelling of the ePB-MSCs

The technique of ^99m^Tc labelling the ePB-MSCs was described in a previous study performed by our group (16). In a first step, stannous chloride powder (Sigma Aldrich, US) was liquefied in sterile basic water (pH 8.5). Then, 0.9–1 x 10^6^ ePB-MSCs were thawed, transferred into culture medium (DMEM supplemented with fetal bovine serum) and centrifuged. The obtained cell pellet was resuspended in 4 mL saline and mixed with 5 mg SnCl_2_ and 45 ± 5 mCi (1665 ± 185 MBq) of freshly eluted ^99m^Tc04- (^99m^Pertechnetate) from a molybdenum generator (GE health care, Eindhoven, The Netherlands). The preparation was subsequently incubated for 30 min at room temperature before centrifugation. The cell pellet was washed and centrifuged again. The final cell pellet was resuspended in 1 mL DMEM, a cell count was performed and the post-labelling viability of the ePB-MSCs was determined using trypan blue staining. At each centrifugation step, the radioactivity of the supernatant was measured in a dose calibrator and used to determine the labelling efficiency.

### Treatment

The patient received the treatment on the day of the experiment. First, the dog was sedated with dexmedetomidine (12–25 μg/kg IM). Next, the anesthesia was induced using propofol (dosage on effect) and maintained with isoflurane 1.2–1.4% (on effect) in 100% oxygen after endotracheal intubation. The dog was positioned in sternal recumbency on a table over the gamma camera before being administered an intravenous injection with ^99m^Tc-labelled ePB-MSCs through a 22-gauge catheter in one of the cephalic veins.

### Imaging protocol

**Figure 2:**
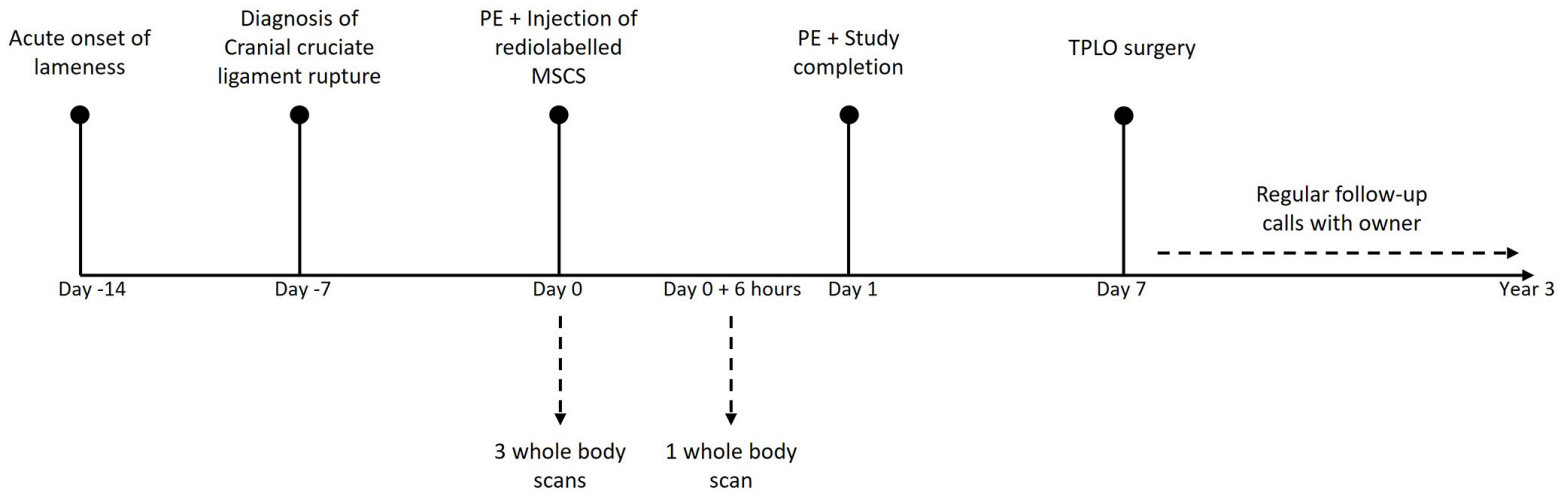
Representative timeline of the experiment. PE, Physical examination; MSC, mesenchymal stem cell; TPLO, tibial plateau levelling osteotomy.

The scintigraphic examination was performed using a two-headed gamma camera, equipped with low energy high resolution collimators (GCA 7200 A; Toshiba). The whole-body scan was obtained with the detectors of the SPECT scanner moving simultaneous dorsally and ventrally from head to tail of the dog over 20 min. During all the acquisitions the dog was kept under general anesthesia. During the first hour, three successive acquisitions were performed for data collection. The first acquisition was started together with the intravenous injection of the radiolabelled ePB-MSCs and the dog was kept in an unchanged position for all scans. Another total body scan, 6 h after the intravenous injection of the radiolabelled ePB-MSCs, was performed under general anesthesia using propofol (dosage on effect) (Figure 2). For each scan, a syringe with a known amount of radioactivity was put next to the dog to calculate the percentage of injected activity (% ID). Care was taken for the dog's re-positioning on the table, to avoid too much spatial deviation on the scans following the first hour scans.

### Image interpretation

The distribution of the labelled ePB-MSCs was first assessed descriptively through the whole body. Consequently, the radioactivity in the regions of interest was quantified in different manually drawn regions of interest (ROI) on the dorsal and ventral view of the whole-body scans (matrix size 512 x 1,024) using the free-hand region of interest tool of a DICOM viewing software platform (Hermes MultiModalityTM, Nuclear Diagnostics, Sweden). A geometric mean of dorsal and ventral activity for each time point and each ROI was calculated to compensate for attenuation. To keep shape and sizes (number of pixels) of the different organ ROI's uniform, a ROI template was created per dog and used for the different time points. A specific organ ROI was drawn on the image on which the organ was best delineated and thereafter used for the other images. Relative uptake of MSCs in the lesion joint was compared with the control joint.

### Clinical follow-up

Seven days after the experiment, a tibial plateau levelling osteotomy (TPLO) was performed by an independent surgeon to stabilize the injured joint of the dog. In order to document the clinical outcome, the investigator regularly contacted the owner of the dog until 3 years after the study completion (Figure 2).

## Results

### Labelling efficiency, post-labelling viability, and injected dose

The labelling efficiency was 70.98% and 1,035,000 ePB-MSCs with a dose of 18.31 mCi ^99m^Tc were injected with a post-labelling cell viability of 90.82% (Table 1).

**Table 1:** Percentage of decay corrected injected dose observed in the different organs 10, 60 min, and 6 h following intravenous injection of the radiolabelled ePB-MSCs.

	**10 min (% ID)**	**60 min (% ID)**	**6 h (% ID)**
Heart	0.58	0.44	0.39
Lung	1.81	1.57	1.29
Liver	10.62	10.36	9.86
Spleen	0.71	0.66	0.66
Bladder	0.38	0.48	0.51

### Safety

The parameters rectal temperature, respiratory rate, heart rate, mucosal membranes, capillary refill time, body conditions score, mentation and hydration were in the physiological range at all-time points of observation. No abnormal general clinical signs were observed and no (serious) adverse events or suspected adverse drug reactions were observed during the study.

### Biodistribution

Following intravenous injections of the of the labelled ePB-MSCs into the cephalic vein of the dog, presence was predominantly observed in the heart, lung, liver, spleen and bladder. The highest uptake was seen in the liver that decreased over time. An initial uptake was present in the heart, lung and spleen and this uptake progressively decreased over time. Finally, a progressively increasing uptake was observed in the bladder until 6 h post injection (Table 1).

**Figure 3:**
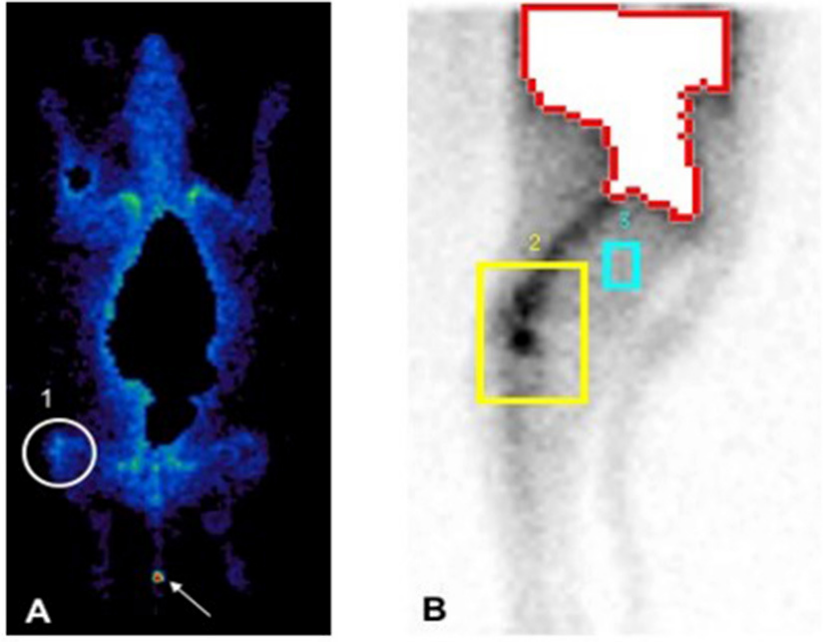
Total body scan 60 minutes **(A)** and lateral scan of the hind limb 6 hours **(B)** following intravenous injection of the radiolabelled ePB-MSCs. The white circle (1) and yellow square (2) shows the higher activity in the stifle joint with ruptured CCL, the blue square (3) represents background correction and the arrow shows a local hotspot in the tail of the dog.

Approximately 40.61% higher radioactive uptake was present in the injured knee (background corrected counts per pixels: 15,076) in comparison to the healthy contralateral knee (background corrected counts per pixels: 8,953) 6 h post-injection (Figure 3). Furthermore, radioactive uptake was observed in the tail of the dog indicating a local hotspot which coincided with the reporting of the owner that a door had accidentally been closed on the tail of the dog.

### Clinical outcome

The TPLO surgery was a success and after a graduate exercise program of 8 weeks, the dog was able to return to it's previous amount of exercise. During the 3 years following the experiment and the surgery, the dog remained clinically sound while continuing to have regular exercising and no lameness was reported by the owner in any of the limbs. Moreover, no cranial cruciate ligament rupture occurred in the contralateral limb.

## Discussion

To the authors' knowledge, this is the first study to investigate the distribution pattern of systemically administered xenogeneic ePB-MSCs in a naturally occurring cruciate ligament lesion in a dog.

In line with the observation in recent studies of our group (16), no clinical abnormalities or ectopic tissue formations were detected throughout the study. Additionally, after study completion, the investigator remained in contact with the dog owner and no clinical abnormalities were reported since. Consequently, the administration of equine xenogeneic MSCs results in a positive safety profile.

Following the injection, an important radiopharmaceutical uptake was mainly present in the liver of the dog. A lower radioactive uptake was also seen in the heart, lung, spleen, kidneys and bladder. The radioactive accumulation in these organs was comparable to what was observed in the studies reported in previous biodistribution studies performed by our group (16). In contrast to other groups studying the distribution of labelled mesenchymal stem cells in dogs and rabbits, no initial pulmonary trapping was seen in the current case (15, 18, 19). The absence of pulmonary entrapment was also seen in a previous study performed by our group and might be explained by the use of a different source of= MSCs and a lower number of injected MSCs (16). Similar to these studies the radioactive accumulation in the bladder slightly increased post-injection and is probably caused by a dissociation of the ^99m^Tc label from the stem cells after cell death, because intact MSCs cannot pass the glomerular filtration.

The current study is the first to describe distribution pattern of IV administration of xenogeneic ePB-MSCs in a dog with a naturally occurring joint lesion and showed a 40.61% higher uptake in the affected joint in comparison with the healthy contralateral joint. Furthermore, a local hotspot was seen in a part of the tail which had previously been injured. These observations are in line with the results from another study performed by our group (article under review) where homing to the joints in an induced OA model was observed and gives more insights on the pharmacokinetics of the ePB-MSCs. In this regard, it has been reported that MSCs possess homing capacities indicating they can be recruited, locally and systemically, to sites with tissue damage. After intravenous injection, MSCs preferentially reach damaged tissue through systemic circulation (20, 21). MSCs exit systemic circulation at the target site by traditional methods of diapedesis followed by interstitial migration (21).

During the regular follow-up calls with the owner up to 3 years following the experiment, it was reported that the dog was clinically sound and that no cranial cruciate ligament lesion occurred in the contralateral limb. In this regard, Muir et al. (22) reported that contralateral cranial cruciate ligament ruptures occur in 54% of the dogs with a Median survival time to subsequent contralateral lesion of 947 days.

The current case report has several limitations. Indeed, the cells were only tracked until 6 h following injection to reduce the number of anesthesia's performed on the dog. Additional studies in a higher number of animals and with a longer tracking period are needed to confirm the observations seen in this experiment. Furthermore, given the short duration of the study, no long-term safety evaluation was performed.

The results obtained in this experiment can help to further elucidate the mode of action of the stem cells by which homing might be a part. Indeed, it was shown that the MSCs home to the injured joints. However, further research is warranted to investigate the precise mode of action of the xenogeneic MSCs.

## Data availability statement

The raw data supporting the conclusions of this article will be made available by the authors, upon reasonable request.

## Ethics statement

BIVMB Ethics Committee as approved by the Flemish government (permit number: LA1700607). Written informed consent was obtained from the owners for the participation of their animal in this study.

## Author contributions

CB and JSp conceived and planned the design of the study. 99mTc labelling was performed by CB, ED, and GP. IV ePB-MSCs administration, animal handling, and scintigraphy was performed by CB, YX, and KP. CB and KP analysed and quantified the scintigraphy images. CB, GP, and KP interpreted the data, managed the literature research, and wrote the first draft of the manuscript. JSp and JSa provided supervision and critical review of the manuscript. All authors contributed and approved the final version of the manuscript.

## Funding

The authors declare that this study received funding from Boehringer Ingelheim Veterinary Medicine Belgium (BIVMB) NV. The funder had the following involvement with the study: study design, data collection and analysis, decision to publish, and preparation of the manuscript.

## Conflict of interest

CB, ED, and GP were all employed by BIVMB. ED, CB, and JSp are the inventors of a pending patent covering the 99mTc labelling of ePB-MSCs owned by GST (EP19200152.7). The content of this manuscript contains a stem cell product under development owned by BIVMB. The remaining authors declare that the research was conducted in the absence of any commercial or financial relationships that could be construed as a potential conflict of interest.

## Publisher's note

All claims expressed in this article are solely those of the authors and do not necessarily represent those of their affiliated organizations, or those of the publisher, the editors and the reviewers. Any product that may be evaluated in this article, or claim that may be made by its manufacturer, is not guaranteed or endorsed by the publisher.

## References

[1] Anderson KL Zulch H O'Neill DG Meeson RL Collins LM. Risk factors for canine osteoarthritis and its predisposing arthropathies: a systematic review. Front Vet Sci. (2020) 7:220. 10.3389/fvets.2020.00220 32411739 PMC7198754

[2] Pettitt RA German AJ. Investigation and management of canine osteoarthritis. In Pract. (2015) 37:1–8. 10.1136/inp.h5763

[3] Baraniak PR McDevitt TC. Stem cell paracrine actions and tissue regeneration. Regen Med. (2010) 5:121–43. 10.2217/rme.09.74 20017699 PMC2833273

[4] Nöth U Steinert AF Tuan RS. Technology Insight: adult mesenchymal stem cells for osteoarthritis therapy. Nat Clin Pract Rheumatol. (2008) 4:371–80. 10.1038/ncprheum0816 18477997

[5] Guercio A Di Marco P Casella S Cannella V Russotto L Purpari G. Production of canine mesenchymal stem cells from adipose tissue and their application in dogs with chronic osteoarthritis of the humeroradial joints. Cell Biol Int. (2012) 36:189–94. 10.1042/CBI20110304 21936851

[6] Sasaki A Mizuno M Mochizuki M Sekiya I. Mesenchymal stem cells for cartilage regeneration in dogs. World J Stem Cells. (2019) 11:254–69. 10.4252/wjsc.v11.i5.254 31171954 PMC6545524

[7] Guercio A Di Bella S Casella S Di Marco P Russo C Piccione G. Canine mesenchymal stem cells (MSCs): characterization in relation to donor age and adipose tissue-harvesting site. Cell Biol Int. (2013) 37:789–98. 10.1002/cbin.10090 23505013

[8] Sancak G Özen I Bayraktaroglu A Alev A Can A Pinar P. Characterization of mesenchymal stem cells isolated from the adipose tissue of young and old dogs. Ankara Üniversitesi Veteriner Fakültesi Dergisi. (2016) 63:297–302. 10.1501/Vetfak_0000002743

[9] De Schauwer C Meyer E Van de Walle GR Van Soom A. Markers of stemness in equine mesenchymal stem cells: a plea for uniformity. Theriogenology. (2011) 75:1431–43. 10.1016/j.theriogenology.2010.11.008 21196039

[10] Spaas JH De Schauwer C Cornillie P Meyer E Van Soom A Van de Walle GR. Culture and characterisation of equine peripheral blood mesenchymal stromal cells. Vet. J. (2013) 195:107–13. 10.1016/j.tvjl.2012.05.006 22717781

[11] Tsai S-Y. Intra-articular transplantation of porcine adipose-derived stem cells for the treatment of canine osteoarthritis: a pilot study. World J Transplant. (2014) 4:196. 10.5500/wjt.v4.i3.196 25346893 PMC4208083

[12] Daems R Hecke L Van Schwarzkopf I Depuydt E Broeckx SY David M. A Feasibility study on the use of equine chondrogenic induced mesenchymal stem cells as a treatment for natural occurring osteoarthritis in dogs. Stem Cell Int. (2019) 2019:1–11. 10.1155/2019/4587594 31281373 PMC6589207

[13] Mei SHJ Haitsma JJ Dos Santos CC Deng Y Lai PFH Slutsky AS. Mesenchymal stem cells reduce inflammation while enhancing bacterial clearance and improving survival in sepsis. Am J Respir Crit Care Med. (2010) 182:1047–57. 10.1164/rccm.201001-0010OC 20558630

[14] Olsen A Johnson V Webb T Santangelo KS Dow S Duerr FM. Evaluation of intravenously delivered allogeneic mesenchymal stem cells for treatment of elbow osteoarthritis in dogs: a pilot study. Vet Comp Orthop Traumatol. (2019) 32:173–81. 10.1055/s-0039-1678547 30873568

[15] Barthélémy I Thibaud J-L de Fornel P Cassano M Punzón I Mauduit D. In vivo stem cell tracking using scintigraphy in a canine model of DMD. Sci Rep. (2020) 10:10681. 10.1038/s41598-020-66388-w 32606364 PMC7327062

[16] Beerts C Brondeel C Pauwelyn G Depuydt E Tack L Duchateau L. Scintigraphic tracking of 99mTechnetium-labelled equine peripheral blood-derived mesenchymal stem cells after intravenous, intramuscular, and subcutaneous injection in healthy dogs. Stem Cell Res Ther. (2021) 12:1–11. 10.1186/s13287-021-02457-9 34256833 PMC8278733

[17] Broeckx S Zimmerman M Aerts D Seys B Suls M Mariën T. Tenogenesis of equine peripheral blood-derived mesenchymal stem cells: *in vitro* versus *in vivo*. J Tissue Sci Eng. (2012) 1–6. 10.4172/2157-7552.S11-001

[18] Meseguer-Olmo L Montellano AJ Martínez T Martínez CM Revilla-Nuin B Roldán M. Intraarticular and intravenous administration of 99MTc-HMPAO-labeled human mesenchymal stem cells (99MTC-AH-MSCS): in vivo imaging and biodistribution. Nucl Med Biol. (2017) 46:36–42. 10.1016/j.nucmedbio.2016.12.003 28013120

[19] Spriet M Hunt GB Walker NJ Borjesson DL. Scintigraphic tracking of mesenchymal stem cells after portal, systemic intravenous and splenic administration in healthy beagle dogs. Vet Radiol Ultrasound. (2015) 56:327–34. 10.1111/vru.12243 25582730

[20] Bagno LL Salerno AG Balkan W Hare JM. Mechanism of action of mesenchymal stem cells (MSCs): impact of delivery method. Expert Opin Biol Ther. (2022) 22:449–63. 10.1080/14712598.2022.2016695 34882517 PMC8934282

[21] Ullah M Liu DD Thakor AS. Mesenchymal stromal cell homing: mechanisms and strategies for improvement. iScience. (2019) 15:421–38. 10.1016/j.isci.2019.05.004 31121468 PMC6529790

[22] Muir P Schwartz Z Malek S Kreines A Cabrera SY Buote NJ. Contralateral cruciate survival in dogs with unilateral non-contact cranial cruciate ligament rupture. PLoS ONE. (2011) 6:e25331. 10.1371/journal.pone.0025331 21998650 PMC3187768

